# Validated method to measure yakuchinone A in plasma by LC-MS/MS and its application to a pharmacokinetic study in rats

**DOI:** 10.1186/1752-153X-8-2

**Published:** 2014-01-14

**Authors:** Feng Chen, Hai-Long Li, Yin-Feng Tan, Wei-Wei Guan, Yong-Hui Li, Jun-Qing Zhang

**Affiliations:** 1 School of Pharmacy, Hainan Medical University, Hainan Provincial Key Laboratory of R&D of Tropical Herbs, Haikou 571101, China

**Keywords:** Yakuchinone A, LC-MS/MS, Matrix effects, HCOOH, Pharmacokinetic study, SuoQuan capsules

## Abstract

**Background:**

Yakuchinone A has a plethora of beneficial biological effects. However, the pharmacokinetic (PK) data of yakuchinone A still remain unknown so far. Furthermore, the quantification of yakuchinone A in biological samples has not been reported in the literature. Therefore, in the present study we aimed to develop a new method for the fast, efficient and accurate assessment of yakuchinone A concentration in plasma, as a means for facilitating the PK evaluation of yakuchinone A.

**Results:**

A liquid chromatography-electrospray ionization-tandem mass spectrometry (LC-ESI-MS/MS) method was developed and validated for the determination of yakuchinone A in rat plasma. Mass spectrometric and chromatographic conditions were optimized. Plasma samples were pretreated by protein precipitation with methanol. LC separation was performed on a Phenomenex Luna C18 column with gradient elution using a mobile phase consisting of methanol–water containing 0.5 mM formic acid (HCOOH) at a flow rate of 0.28 mL/min. ESI-MS spectra were acquired in positive ion multiple reaction monitoring mode (MRM). The precursor-to-product ion pairs used for MRM of yakuchinone A and yakuchinone B were m/z 313.1 → 137.0 and 311.2 → 117.1, respectively. Low concentration of HCOOH reduced the ion suppression caused by matrix components and clearly improved the analytical sensitivity. Yakuchinone A showed good linearity over a wide concentration range (r > 0.99). The accuracy, precision, stability and linearity were found to be within the acceptable criteria. This new method was successfully applied to analyze the rat plasma concentration of parent yakuchinone A after a single oral administration of SuoQuan capsules. Low systemic exposure to parent yakuchinone A was observed.

**Conclusion:**

The proposed method is sensitive and reliable. It is hoped that this new method will prove useful for the future PK studies.

## Background

Yakuchinone A [1-(4′-hydroxy-3′-methoxyphenyl)-7-phenyl-3-heptanone] was isolated from the fruits of *Alpinia oxyphylla* Miq. (Zingiberaceae) [1]. In East Asian traditional medicine, these edible fruits are widely used for treating dyspepsia, diarrhea, polyuria, and gastralgia [2]. Pharmacological studies both *in vitro* and *in vivo* have confirmed that yakuchinone A has a plethora of beneficial biological effects. Yakuchinone A strongly inhibits prostaglandin biosynthesis *in vitro*[3,4]. Yakuchinone A has inhibitory effects on phorbol ester-induced inflammation and skin carcinogenesis in mice and oxidative stress *in vitro*[5]. Yakuchinone A induces apoptotic death in cultured human promyelocytic leukemia cells [6,7]. Moreover, yakuchinone A inhibits the expression of cyclooxygenase-2 and of inducible nitric oxide synthase, as well as the expression of tumor necrosis factor-alpha mRNA in mouse skin [5,8]. These findings demonstrate that this compound has anti-inflammatory properties. In addition, the compound suppresses the spontaneous calcium spikes and contraction in isolated portal veins of mice [9]. However, in our lab, we have found that yakuchinone A significantly induces the contraction of rat detrusor muscles *in vitro*.

Pharmacokinetics is an integral part of pharmacological research of botanical product and the appropriate PK properties are a prerequisite for the compound to play a role pharmacologically. However, the PK data of yakuchinone A still remain unknown so far. To the best of our knowledge, the quantification of yakuchinone A in biological samples has not been reported in the literature. Thus, we sought to develop a new method for the fast, efficient and accurate assessment of yakuchinone A concentration in plasma, as a means for facilitating the PK evaluation of yakuchinone A. Here we report the development and full validation of a rapid and sensitive method based on high performance liquid chromatography with tandem mass spectrometry (LC-MS/MS) for determination of yakuchinone A in rat plasma, as well as the use of this method to analyze samples obtained during a single oral PK study in Sprague-Dawley (SD) rats.

## Experimental

### Chemicals and reagents

Reference standards of yakuchinone A and B (used as internal standard, IS) were purchased from Chenfun Medical Technology (Shanghai) Co., Ltd. (Shanghai, China). The purity of these compounds was > 98%. HPLC-grade methanol and acetonitrile were products of Sigma-Aldrich (St Louis, MO, USA). HPLC-grade HCOOH was purchased from Aladdin Industrial Inc. (Shanghai, China). HPLC-grade water was prepared by double-distillation of deionized water. The other chemical reagents of analytical grade or better were obtained from Hainan YiGao Instrument Co., Ltd (Haikou, China). The utilized SuoQuan capsules are commercially available *A. Oxyphyllae* Fructus products. The SuoQuan capsules were purchased from Hansen Pharm. (lot no.110602, expiration: 2013/05; Chinese SFDA ratification no.Z19991039; Yiyang, Hunan Province, China) and each capsule contains 0.3 g of solid.

The main constituents occurring in SuoQuan capsules have been measured by LC-MS/MS [10]. Each capsule contained the following amounts of phytochemicals: nootkatone (142 μg), yakuchinone A (162 μg), yakuchinone B (7.28 μg), oxyphyllacinol (12.7 μg), boldine (63.0 μg), norisoboldine (388 μg), linderane (3.90 μg), isolinderalactone (2103 μg), atractylenoide III (1.73 μg), tectochrysin (8.00 μg), izalpinin (1.70 μg), chrysin (5.12 μg), apigenin-4′,7-dimethyl ether (11.5 μg) and kaempferide (1.25 μg), respectively.

### Animal study

All rat experiments were performed in accordance with the Institutional Animal Care and Use Committee at the Hainan Medical University (Haikou, China), as well as the Guidance for Ethical Treatment of Laboratory Animals (The Ministry of Science and Technology of China, 2006). Male SD rats (310–330 g) were purchased from DongChuang Laboratory Animal Service Department (Changsha, China). Rats were maintained under controlled temperature of 24 ± 2°C and relative humidity of 60% ± 10% with a 12-h light/dark cycle. Commercial rat chow was available *ad libitum* except for an overnight fasting period before dosing. All rats had free access to water.

For the PK study, three rats received a single oral ingestion of SuoQuan capsules at 5.7 g/kg via gavage. Before use, the drugs enclosed in the hard “shell” were suspended in 0.5% w/v sodium carboxymethyl cellulose. Serial blood samples (~ 0.3 ml each at 5, 10, 20 and 30 min, 1, 2, 4, 6, 8, 11 and 24 h post-dosing) were collected in heparinized tubes. The blood samples were centrifuged to obtain the plasma fractions that were frozen at –70°C until analysis.

Plasma PK parameters were estimated by a noncompartmental method using the Kinetica 2000 software package (version 3.0; Innaphase Corp., Philadelphia, PA). The *C*_max_ and the *T*_max_ were observed values with no interpolation. The area under concentration-time curve up to the last measured time point (AUC_0→t_) was calculated by the trapezoidal rule. The AUC_0→∞_ was generated by extrapolating the AUC_0→t_ to infinity.

### Plasma sample preparation

Plasma samples (50 μL) were treated with 150 μL methanol containing the IS (300 ng/mL). The mixture was mixed by vortex-shaking for 10 min and centrifuged at 13, 000 × g for 10 min. 10 μL of supernatant were applied to LC -MS/MS analysis.

### LC-MS/MS conditions

The LC-MS/MS system consisted of an AB-SCIEX API 4000^+^ mass spectrometer (Toronto, Canada) interfaced via a Turbo V ion source with a Shimadzu Prominence UFLC chromatographic system (Shimadzu Corporation, Kyoto, Japan), which is equipped with two LC-20 AD pumps, a model DGU-20A3R degasser unit, a SIL-20A HT autosampler and a CTO-20A column oven. The AB-SCIEX Analyst software packages were used to control the LC-MS/MS system, as well as for data acquisition and processing.

Chromatographic separations of prepared samples were achieved using a phenomenex ® Luna C18 column (5 μm, 2.0 mm i.d × 50 mm) maintained at 40°C. The LC mobile phases (delivered at 0.28 mL/min) included water containing 0.5 mM HCOOH for solvent A and methanol containing 0.5 mM HCOOH for solvent B. A specially designed LC binary gradient elution was performed and the gradient program was as fallows: 0–1 min at 0% B; from 0% B to 80% B in 0.01 min; from 80% B to 100% B in 2.5 min; back to 0% B in 0.01 min; maintained 2.5 min.

**Figure 1:**
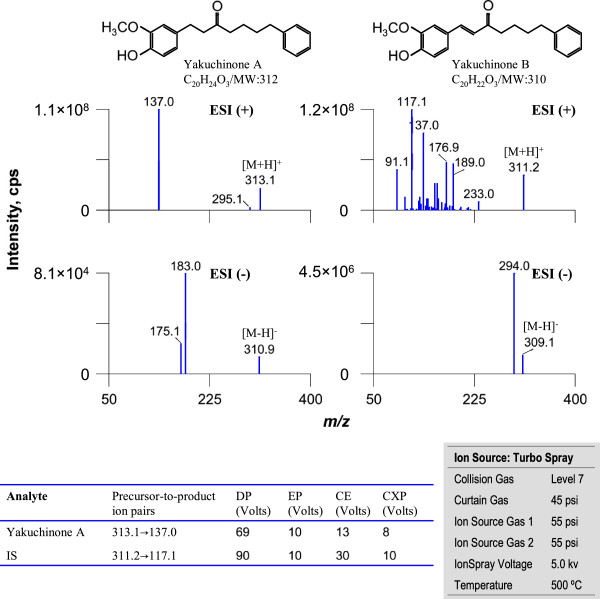
**Typical MS/MS product ion spectra of yakuchinone A and B.** The intensities of the [M + H]^+^or the [M-H]^-^ ions produced in the ESI source were compared for both analytes. The chemical structures and MS/MS conditions are shown on the upper and the lower panel, respectively. The experiment was performed under ***Manually Tuning*** mode by a syringe infusing the standard solution of Yakuchinone A and B (1 μg/ml) at a rate of 5 μL/min.

The mass spectrometer was operated in the positive ion ESI mode with MRM for all the analytes. The pneumatically nebulized ESI spraying was achieved by using inner coaxial nebulizer N_2_ gas (GS1) of 55 psi through a TurboIonSpray probe, a high voltage of + 5.0 kV applied to the sprayer tip, and heated dry N_2_ gas (GS2) of 55 psi at 500°C from two turbo heaters adjacent to the probe. To prevent solvent droplets from entering and contaminating the ion optics, a curtain N_2_ gas of 45 psi was applied between the curtain plate and the orifice. The collision gas (CAD) flow was set at level 7. The precursor-to-product ion pairs (Figure 1) used for MRM of yakuchinone A and B were m/z 313.1 → 137.0 and 311.2 → 117.1, respectively, with a scan time of 30 ms for each ion pair.

### Construction of standard curves

Standard curves were constructed within the plasma concentration range 1 to 2000 ng/mL by plotting the peak area ratios (*Y*) of the analytes to the IS against the corresponding nominal plasma concentrations of the analyte (*X*, ng/mL) and the 1/*X*^2^ was used as a weighting factor.

### Method validation

Assay validation was carried out according to the US FDA guidance on bioanalytical method validation (http://www.fda.gov/downloads/Drugs/Guidances/ucm070107.pdf) to demonstrate that the newly developed bioanalytical method was reliable for the intended applications. The quality control samples were prepared from an independent weighing of the reference standard.

## Results and discussion

### Optimization of mass spectrometric and chromatographic conditions

Positive or negative ionization methods were tested and compared to obtain good specificity and sensitivity for yakuchinone A and IS determination. As shown in Figure 1, positive ESI was found to be more sensitive than negative ESI by infusing an approximately 1 μg/mL standard stock solution of yakuchinone A and IS in acetonitrile using a Harvard infusion pump (Harvard Apparatus, South Natick, MA, USA). During a direct infusion experiment, the mass spectra for yakuchinone A and IS revealed peaks at m/z 313.1 and 311.2, respectively as protonated molecular ions [M + H]^+^. The product ion mass spectrum for yakuchinone A shows the formation of characteristic product ion at m/z 137.0. For IS, the m/z 117.1 fragment was dominant and was therefore used for quantification.

**Figure 2:**
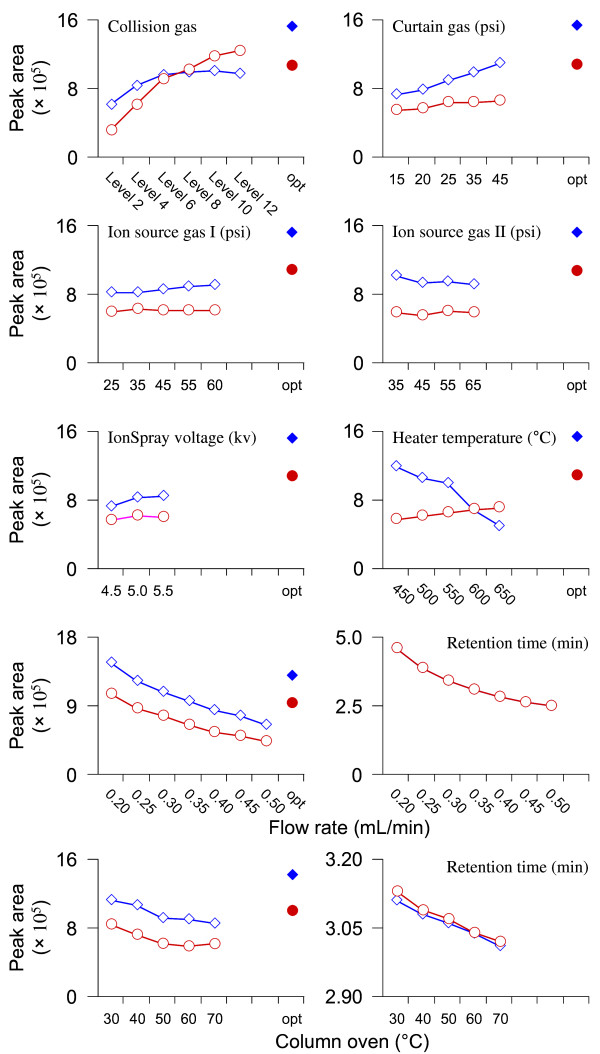
**MS/MS parameters and LC conditions optimization.** For each MS/MS parameter optimization, the other normal LC-MS/MS conditions were used, including ion source parameters (collision gas, level 4; curtain gas, 25 psi; Gas 1, 55 psi; Gas 2, 55 psi; ionspray voltage, 5.5 kv; temperature, 550°C) and LC parameters (flow rate, 0.30 mL/min; column oven temperature, 40°C). Finally, the resulting optimized parameters were combined and the signal intensity of the test yakuchinone A (blue open diamond symbol and blue solid diamond symbol) and B (red open circle symbol and red solid circle symbol) were compared with those of data under premature LC-MS/MS conditions.

On the basis of the optimized ionization mode, the mass spectrometric parameters including the collision gas (CAD), curtain N_2_ gas (CUR, psi), GS1 (psi), GS2 (psi), TurboIonSpray probe voltage (kv), and ion source temperature (°C) were further optimized in order to get the richest relative abundance of precursor-to-product ions. As shown in Figure 2, the parameters CAD, CUR and temperature significantly influenced the peak areas of yakuchinone A and IS. The lower panel of Figure 1 shows the optimized MS/MS compound parameters (left) and ion source parameters (right). At the end, the highest peak area for molecular ion of the analyte was achieved when the optimized compound parameters and ion source parameters were combined (Figure 2).

To achieve the efficient separation of yakuchinone A and IS, different mobile phases (methanol/water system and acetonitrile/water system) were tested. Compared with methanol used as organic phase, the peak shape was widened and the peak area decreased significantly (~ 10-fold). However, the test mobile phase system had little influence on the retention time. Therefore, methanol/water system was chosen as the mobile phase. In addition, as the mobile phase flow rate and column oven temperature increased, the peak area, as well as the corresponding retention time, of yakuchinone A and IS decreased (Figure 2, the bottom panels). The best separation was obtained when gradient elution was performed and column temperature was kept at 40°C using a flow rate of 0.28 mL/min.

### Method validation

#### Specificity and selectivity

**Figure 3:**
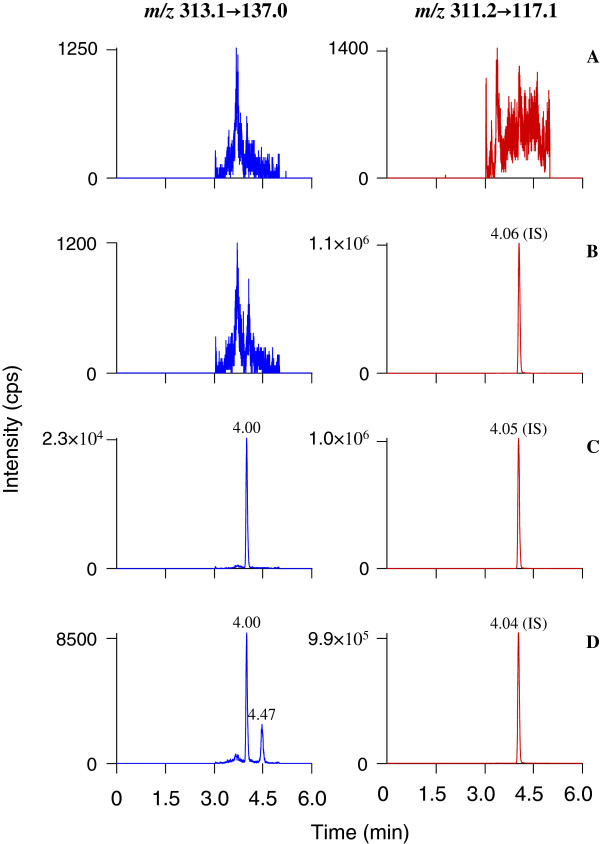
LC-MS/MS chromatograms for a typical blank rat plasma sample (panel A), the same blank plasma sample spiked with yakuchinone B (IS, panel B), a mixture of standard yakuchinone A and IS (panel C), and an IS-spiked plasma sample obtained from a rat 5 min after receiving a single oral dose of Suoquan capsules at 5.7 g/kg (panel D).

Selectivity was investigated by comparing the chromatograms of six different blank rat plasma samples with the corresponding spiked plasma samples with known concentrations of the analytes. The retention times for yakuchinone A and IS were about 4.00 and 4.05 min, respectively. The limit of detection (LOD) and limit of quantification (LOQ) were the concentrations at which the analyte signal-to-noise ratios were 3:1 and 10:1, respectively. They were achieved by serial dilution of spiked plasma sample solutions using the described LC-MS/MS conditions. The LOD and LOQ of yakuchinone A were 0.05 and 0.5 ng/mL, respectively. A representative chromatogram is illustrated in Figure 3, including blank plasma, spiked plasma sample with analytes in LLOQ level and IS, as well as a plasma sample from rat 5 min after receiving a single oral dose of Suoquan capsules. No peaks from endogenous biological matrix or other source were observed at the same retention time of Yakuchinone A and IS in any of the blank plasma, which suggested that the developed procedure was specific and selective.

### Linearity and LLOQ

The matrix-based calibration curve (*Y* = 0.00202*X* + 0.000608) was linear over the concentration range of 1–2000 ng/mL for yakuchinone A with correlation coefficient of 0.997. The LLOQ samples of six different rat plasma independent of the calibration curves were analyzed. The LLOQ was 1 ng/mL (S/N > 10), with a precision of 5.88% and accuracy of 97.0% for yakuchinone A.

### Precision and accuracy

As shown in Table 1, the newly developed method gave good precision and accuracy with the intra- and inter-day assays. The intra-day accuracy ranged from 85.8% to 102% for yakuchinone A and from 96.3% to 102% for IS; as well as the inter-day accuracy ranged from 88.1% to 101% for yakuchinone A and 96.6% for IS, respectively. The intra- and inter-day precisions were within 0.46%–5.90% for yakuchinone A and 3.23%–3.93% for IS, respectively. The results for both intra- and inter-day accuracy and precision were found to be within the acceptable criteria and allowed the accurate assay of the analytes in rat plasma.

**Table 1:** Precision and accuracy for the yakuchinone A and IS in rat plasma (n = 15, 5 replicates per day for 3 days)

**Nominal conc. (ng/ml)**	**Peak area (× 10**^**4**^**)**											
	**Intra-day**									**Inter-day**		
	**Day 1**			**Day 2**			**Day 3**					
	**Mean ± SD**	**RSD**	**Accuracy**	**Mean ± SD**	**RSD**	**Accuracy**	**Mean ± SD**	**RSD**	**Accuracy**	**Mean ± SD**	**RSD**	**Accuracy**
Yakuchinone A												
4	5.72 ± 0.12	2.11%	101%	5.43 ± 0.16	2.98%	94.1%	5.31 ± 0.31	5.90%	91.5%	5.49 ± 0.27	4.89%	95.4%
80	94.6 ± 1.6	1.71%	102%	90.3 ± 2.5	2.81%	97.7%	93.7 ± 1.7	1.78%	101%	92.9 ± 2.7	2.87%	101%
800	818 ± 6	0.77%	89.6%	784 ± 4	0.46%	85.8%	813 ± 12	1.41%	89.0%	805 ± 17	2.13%	88.1%
IS												
80	49.3 ± 1.7	3.52%	102%	48.7 ± 1.6	3.30%	101%	46.7 ± 1.5	3.23%	96.3%	48.2 ± 1.9	3.93%	99.6%

### Extraction recovery and matrix effect

Extraction efficiencies and matrix effects were examined in quintuplicate by comparing analyte peak areas of across three different sample sets [11,12]. In set 1, analytes were dissolved in matrix component-free reconstitution solvent. In set 2, analytes were added into five different lots of pre-extracted plasma from untreated rats. In set 3, analytes were added to plasma from untreated plasma and then extracted. The absolute matrix effect and extraction recovery were calculated as follows:

Absolute matrix effect = (Mean peak area)_set 2_ ⁄ (Mean peak area)_set 1_

Extraction efficiency = (Mean peak area)_set 3_ ⁄ (Mean peak area)_set 2_

This post-extraction spike method proposed by Matuszewski et al. [11] provides a quantitative understanding of the level of matrix effect observed for specific analytes. In the present study, the extraction recoveries of the quality control samples for yakuchinone A and IS are summarized in Table 2. The extraction recovery ranged from 72% to 89% for yakuchinone A and 59% for IS, respectively, which suggested that the extraction recovery of both analytes was efficient, consistent and reproducible. Meanwhile, Table 2 shows the results of matrix effects for both analytes. The average matrix effects at three different concentrations were 86.3–98.5% for yakuchinone A and 112% for IS. However, we could not conclude whether the unseen biological matrix significantly influenced the ionization of analytes and IS because the mobile phases were added with low concentration of HCOOH (0.5 mM), which actually impacted the matrix effects, ESI efficiency and capacity [12-16].

**Table 2:** Matrix effect and extraction recovery for the yakuchinone A and IS in rat plasma (n = 5)

**Nominal conc. (ng/ml)**	**Peak area (× 10**^**4**^**)**									
	**Set 1**		**Set 2**		**Set 3**		**Matrix effect**		**Extraction efficiency**	
	**Mean ± SD**	**RSD**	**Mean ± SD**	**RSD**	**Mean ± SD**	**RSD**	**Mean (%)**	**RSD (%)**	**Mean (%)**	**RSD (%)**
Yakuchinone A										
4	7.45 ± 0.15	2.04%	6.43 ± 0.04	0.70%	5.18 ± 0.12	2.28%	86.3	1.95	80.5	2.30
80	113 ± 6	5.05%	111 ± 3	2.59%	79.9 ± 1.2	1.56%	98.5	6.95	71.7	2.89
800	930 ± 61	6.56%	884 ± 19	2.11%	789 ± 12	1.50%	95.5	8.54	89.2	1.19
IS										
80	80.7 ± 7.8	9.65%	89.7 ± 1.9	2.16%	52.7 ± 2.5	4.66%	112	9.30	58.8	3.68

**Figure 4:**
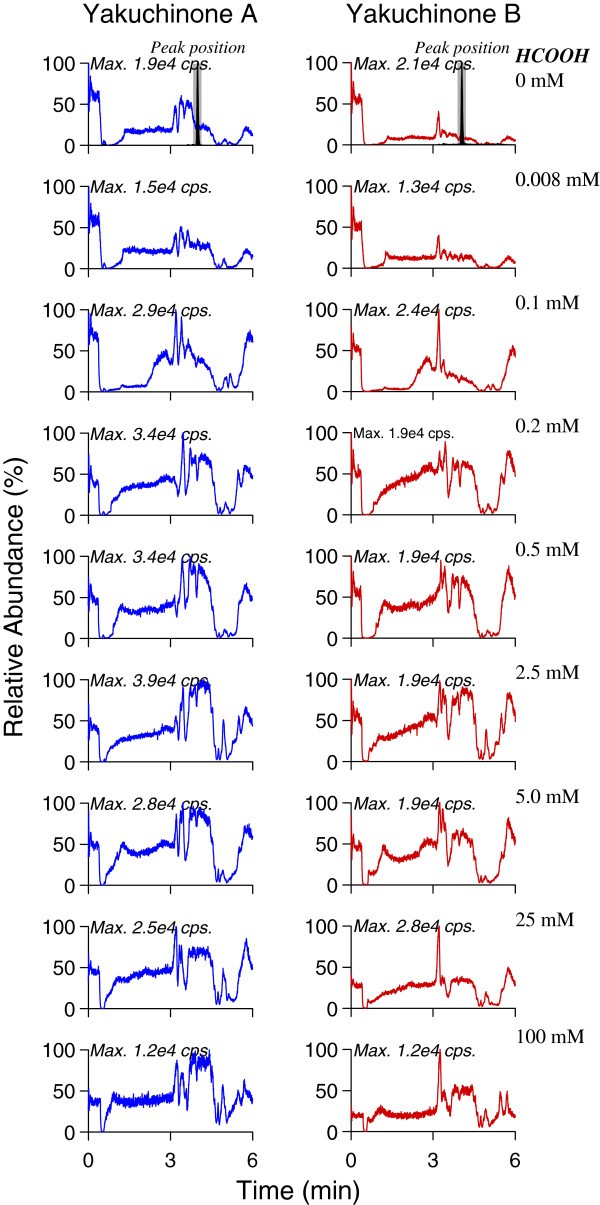
Comparison of infusion chromatograms in the positive ESI ion mode using the mobile phases modified with different concentration of HCOOH under the gradient elution condition.

**Figure 5:**
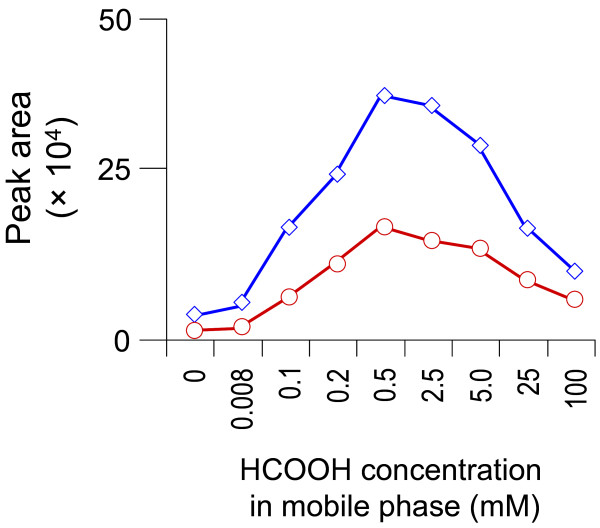
Effect of HCOOH mobile phase concentration on the yakuchinone A (blue open diamond symbol) and B (red open circle symbol) signal intensity in positive-ion mode.

On the other hand, we employed a post-column infusion method to assess the matrix in order to achieve the direct visualization of the nature, chromatographic profile and the extent of the interference caused by matrix components [17,18]. The post-column infusion method provides a qualitative assessment of matrix effects, identifying chromatographic regions most likely to experience matrix effects. Figure 4 shows the infusion chromatograms obtained in the positive ESI ion mode, exhibiting the matrix effect of methanol-precipitated rat plsma on the response to post-column infusion of yakuchinone A and IS. The co-eluting matrix components caused significant ion suppression of both yakuchinone A and IS (upper panel, HCOOH free) and the signal intensity could not come back to the initial level at the end of the elution time program. Therefore, the peak areas of both analytes were diminished after multiple injections under optimized LC-MS/MS conditions. However, the inclusion of HCOOH in the LC mobile phase reduced suppression of the Yakuchinone A and IS signal in a concentration-dependent manner (Figure 4). Concentrations of HCOOH up to 0.1 mM had minimal effect, while concentrations of 0.5-100 mM resulted in comparable matrix effect reduction. The best result appeared to be achieved with the 0.5 mM HCOOH-modified mobile phase, which exhibited significant matrix suppression only during a 1-minute window early in the run (Figure 5).

### Stability

The stabilities of yakuchinone A and IS tested to reflect situations likely to be encountered during actual sample handling and analysis. As summarized in Table 3, the errors in yakuchinone A and IS peak area values for all the test samples were between 1.49% and 13.2% of nominal, well within the limits of acceptability (i.e., not exceeding ± 15%). These findings demonstrate that the yakuchinone A and IS were all acceptably stable under the tested conditions.

**Table 3:** Stability of the yakuchinone A and IS in rat plasma (n = 5)

	**Peak area (× 10**^**4**^**)**					
	**Short-term stability (4 h at room temperature)**		**Autosampler stability (8 h at room temperature)**		**Freeze-thaw stability (3 cycles)**	
	**Mean ± SD**	**RSD**	**Mean ± SD**	**RSD**	**Mean ± SD**	**RSD**
Yakuchinone A						
4	5.04 ± 0.18	3.59%	5.61 ± 0.21	3.72%	5.18 ± 0.13	2.60%
80	79.7 ± 1.8	1.49%	96.4 ± 1.9	2.01%	80.4 ± 7.9	9.84%
800	829 ± 82	9.86%	834 ± 18	2.22%	801 ± 106	13.2%
IS						
80	49.5 ± 1.5	3.04%	51.1 ± 1.7	3.35%	45.7 ± 4.5	9.76%

### Pharmacokinetic application

Finally, the newly developed method was applied to rat PK study of yakuchinone A. In a pilot rat study, yakuchinone A, boldine, norisoboldine and isolinderalactone could be detected in the plasma after a single oral administration of SuoQuan capsules at 5.7 g/kg. In the current study, we focused on the bioanalytical analysis of yakuchinone A, and therefore, the yakuchinone B was used as the internal standard. We found that plasma parent yakuchinone A observed only up to 0.5 h after dosing and the concentrations were quite low with the mean *C*_max_ at 4.62 ng/mL. The plasma parent yakuchinone A peaked at 0.083 h postdose and declined rapidly (mean MRT value, 0.63 h). The mean AUC_0→t_ value was 0.72 h · ng/mL. Meanwhile, the significant individual differences were observed. Like the situation of curcumin [19,20], parent yakuchinone A exhibits a low systemic exposure in rats and the absorbed yakuchinone A may undergo rapid first-pass elimination.

## Conclusion

Here, we developed and validated an analytical method based on LC-MS/MS to measure yakuchinone A concentration in rat plasma treated by a simple protein precipitation procedure. The newly developed method was simple, sensitive and reliable. Mass spectrometric and chromatographic conditions were optimized. Low concentration of HCOOH reduced the ion suppression caused by matrix components and clearly improved the analytical sensitivity. This new method was successfully applied to analyze the rat plasma concentration of parent yakuchinone A after a single oral administration of SuoQuan capsules, an herbal medicine containing the fruits of *A. oxyphylla* Miq. Low systemic exposure to parent yakuchinone A was observed. It is hoped that this new method will be useful for the future PK studies of SuoQuan capsules.

## Abbreviations

HCOOH: Formic acid; LC-ESI-MS/MS: Liquid chromatography-electrospray ionization-tandem mass spectrometry; MRM: Multiple reaction monitoring mode; PK: Pharmacokinetic.

## Competing interests

The authors declare that they have no competing interests.

## Authors’ contributions

CF and LHL were the primary contributors to this manuscript. CF and LHL were responsible for preparing the first draft of the manuscript and performed most of the experimentation and analysis while also being involved heavily in data acquisition and interpretation. ZJQ was involved in design of the experiments and provided critical advice on operation of the analytical equipment due to previous expertise. LYH, GWW and TYF had a significant role in development of the experiments and interpretation of results. All authors read and approved the final manuscript.
